# Two Cases of Fracture of the Scull; with Remarks. To Which Is Added a Case of a Wound of the Head That Terminated Fatally; with an Account of the Appearances on Dissection

**Published:** 1787

**Authors:** Edward Ford

**Affiliations:** Surgeon of the Westminster General Dispensary


### VII. *Two Cases of Fracture of the Scull; with Remarks. To which is added a Case of a Wound of the Head that terminated fatally; with an Account of the Appearances on Dissection. Communicated in a Letter to Dr.* Simmons, *F. R. S. by Mr.* Edward Ford, *Surgeon of the Westminster General Dispensary.*

### C A S E I.

ON the 25th of June last, T. S. a healthy boy, aged thirteen years, and living at No. 96, in Oxford Street, fell from a one-pair-of-stairs window upon the pavement. He was immediately taken up, senseless, and I saw him about twenty minutes after the accident, when he was quite stupid, comatose, and bleeding at the ears and nose. His head was immediately shaved, and I found a swelling projecting on the right side, and situated on that part of the frontal bone which is adjacent to the coronal suture. This was opened to the extent of about two inches, and a fracture discovered, circumscribing a portion of the os frontis, of the size of a shilling, and slightly depressed. This fracture seemed to extend every way; backwards, across the coronal suture to the right parietal parietal bone; on the fame fide towards the os temporis; and on the left fide in the direction of the coronal future.

The hæmorrhage, from this operation, was very profufe, and he partially recovered his fenfes. Some blood was alfo taken from the arm, and opening medicines were adminiftered. The next morning he was better; his pulfe, though oppreffed, was regular, and he was able to articulate a few words.

Confidering the apparent extent of the fracture, I could not remain fatisfied with what had been done; and having obtained the confent of the patient's father to make fome farther opening, I was forry to find, that, by purfuing the line of the fracture on the right fide, acrofs the coronal future, into the parietal bone, and from thence over the fquamous future, cutting through a great portion of the temporal mufcle, I could not follow it on this fide to its full extent, as it perceptibly led towards the bafis of the fcull. On the left fide I laid bare the fracture, and traced it into the coronal future, which I found completely feparated from its indentation. The incifion was then continued in the direction of that future to the diftance of about two inches from the fagittal future.

Confidering now the great extent of the wound, and the little probability of a fuccefsful termination of the cafe, I made no farther opening on the left fide, though the coronal future was plainly felt disjointed, and a feparation of the bones could be perceived quite down to the ear; but contented myfelf with farther enlarging the wound on the right fide, where the principal mifchief feemed to be feated. In profecuting this operation, though two fuch unfortunate circumftances occurred, as the feparation of a future, and a fracture, which, from its courfe, could not be traced to its utmoft extent by the knife, I had the fatisfaction to find there was no extravafated and grumous blood upon the dura mater, where the future was disjointed; the extravafation feeming to be confined to that part of the membrane which had obvioufly received the firft impreffion from the fall.

The benefit derived from this operation was fo great, that feveral days elapfed before any farther affiftance feemed neceffary. Care was taken to keep open the bowels, and the ufual antiphlogiftic regimen was adopted.

The patient gradually recovered his fenfes, fo as to give rational anfwers; but his pulfe was was very quick, and he had frequent ſtartings. This determined me, on the eighth day, to make two perforations with the trephine on the upper part of the os frontis, by which means I was enabled to remove the depreſſed portion of bone. At the part where the trephine was applied, the dura mater looked very well, but ſome grumous blood was found lodged under the depreſſion.

From this time the greater part of the bad ſymptoms diſappeared, and for a fortnight the patient ſeemed to mend, but he did not gain ſtrength. At the end of three weeks colliquative ſweats came on; the ſtartings returned; the diſcharge from the wound became offenſive; and the perioſteum on the lower part of the parietal bone, near the ſquamous ſuture, ſeparated from the cranium.

A liberal uſe of bark and cordials having been found inſufficient to obviate theſe ſymptoms, a new operation ſeemed neceſſary; and in this opinion I had the concurrence and aſſiſtance of Mr. John Howard, Surgeon, and of Mr. Hodges, the patient's Apothecary. Two large perforations, therefore, were now made, and a very conſiderable quantity of fœtid pus was diſcharged from under the ſcull; the dura mater being in ſo ſloughy a ſtate, that the brain became expoſed. The utmoſt care was taken to obviate coſtiveneſs, and the bark was ſtill adminiſtered in large doſes.

In a few days appearances mended. The diſcharge became leſs fœtid, and decreaſed in quantity. The wounds had a florid appearance, and, particularly that on the dura mater, granulated well. The patient's ſtrength daily increaſed; his appetite mended; his ſleep became undiſturbed; his mental faculties were ſoon quite reſtored; and his wound is, at this time, quite cicatriſed.

REMARKS.

The reflections that ariſe from this caſe are very obvious: it is one of the many which elucidate the well-known doctrine, that the miſchief ariſing from fractures of the ſcull is not occaſioned merely by the extent of the fracture, or by the large openings made in the ſcull, either by the nature of the accident, or by the trephine; ſince the inconveniencies in this caſe, as well as in others, were inſtantly removed, when the preſſure (occaſioned firſt by extravaſated blood, and afterwards by pus) was removed from the dura mater. It ſhould likewiſe likewiſe be remarked, that the ſuppuration did not take place in a part immediately contiguous to the fracture, but under a capillary fiſſure at ſome diſtance from it.

Theſe are well-known facts, illuſtrated by daily experience; but I hope this caſe will be of ſtill farther ſervice in adminiſtering comfort to the practitioner in ſimilar ſituations. A blow, ſo violent in its effect as to occaſion a complete ſeparation of a ſuture, has been conſidered as a fatal, or, at beſt, as a very dangerous, accident. To this, in the caſe I have related, was added another circumſtance equally alarming, which was a fracture extending from one ſide of the head to the other, obviouſly in a ſituation where it was impoſſible to give it the advantage of a dilated wound, and of courſe it was incapable of receiving the aſſiſtance of the trephine.

### CASE II.

E. Watſon, a child three years old, fell from a window two ſtories high in St. Martin's Lane. She was taken up immediately, and appeared to have loſt her ſenſes about a minute. The following day ſhe was brought to the Weſtminſter General Diſpenſary. She was at this time healthy healthy and lively, running about, and purſuing her uſual paſtime, as if no injury had been received. Her pulſe was regular, and her appetite good; but there was a ſwelling on the back part of the head, ſeemingly occaſioned by extravaſated blood on the ſcull.

The total abſence of any ſymptom of danger, for nearly three weeks, ſeemed to corroborate the opinion I had formed of the tumor; but at the end of that time, as it did not ſubſide, it was opened, and I diſcovered a fracture, without depreſſion, extending from one parietal bone to the other, acroſs the ſagittal ſuture.

Two table-ſpoonfuls of clear lymph were diſcharged from the wound. The dura and pia mater were both ruptured; and a ſmall portion of the brain was compreſſed between the edges of the fracture, which were ſeparated from each other to a conſiderable diſtance. The fracture was traced to its extremities, about three inches and a half in length; and there was no appearance of grumous blood or of matter preſſing on the brain.

The only circumſtance in the treatment of the wound worth noticing was, that for four or five days there was a diſcharge of clear ſerous fluid, ſo copious as to make it neceſſary to change the bandages every two hours. It was not that bloody ferum which ufually moiftens the dreffings after an operation, but it was perfectly limpid, feeming to be an increafed fecretion from the furface of the brain.

The patient recovered quickly; and the feparation of the bone is ftill to be felt.

REMARKS.

Injuries of the head, producing extravafation of blood on the cranium, are well known to bear fuch a refemblance to fractures, with depreffion, that the moft accurate touch is liable to deception. From the prefent cafe it would appear, that the total abfence of any bad fymptom is not a fufficient reafon to forbear an examination of the tumor, when a fufficient time has been given for abforption: for though every circumftance in this cafe proved favourable, it is reafonable to fuppofe, that, had the operation been longer deferred, fuch fymptoms might have taken place as would have rendered the cure very uncertain.

This cafe, like the preceding one, is a proof of the little injury done to the conftitution by fractures of the fcull, when no compreffion takes place on the brain or its membranes.

*Case of a Wound of the Head that terminated fatally; with an Account of the Appearances on Dissection.*

A poor boy, about six years old, as he was standing on a chair, fell backwards on a glass bottle, and received a wound on the back part of his head. At the time of the accident, and when I saw him, soon after it, he was perfectly sensible.

The scull was bare, and I thought, upon examining with the probe, that it was fissured. The wound was, therefore, dilated, and I found that it was situated on the upper angle of the occipital bone near the lambdoidal suture; what I had conceived to be a fissure appearing now to be, as it were, an incised wound, on the bone, made by the glass.

The second day after the accident, a fever coming on, his head was shaved, but no marks of injury appeared, except a slight elevation of the integuments on the left parietal bone, at which part an incision was instantly made, but without discovering any injury of the scull.

The patient was not delirious; neither had he any comatose symptoms, or sickness, or bleeding from the ears or nose. His appetite was good, but he was very reftlefs and uneafy, jumping up continually from the bed. On the fourth day an eryfipelas began on the forehead, and daily increafed, fpreading to his face, and from thence to the breaft; his pulfe at the fame time being very weak and rapid.

During the firft ten days the antiphlogiftic plan was fteadily purfued. He was bled; opening medicines were adminiftered; and afterwards antimonial wine, with thebaic tincture.

During the laft four days of his life rigors came on in quick fucceffion, and the eryfipelas continued to fpread, accompanied with great fymptoms of debility. The bark was now given; his medicines and diet were of a more cordial kind; and blifters were applied to his back and legs.

Mr. Adair Hawkins, and Mr. John Howard, faw this patient with me, and contributed their affiftance.

On the fourteenth day he died, and the following day I had an opportunity of opening the body. The pericranium adhered every where to the fcull, but its attachment feemed lefs firm than ufual. In fawing through the cranium, wherever the faw happened to wound the meninges ninges of the brain a clear water iſſued forth, and, upon removing the arch of the cranium, about an ounce more of the ſame fluid was collected. The dura mater was every where in a healthy ſtate, adhering ſtrongly to the ſcull; and the wound made on the occipital bone had not penetrated through its ſubſtance.

There was nearly a table-ſpoonful of water in the ventricles of the brain; and, on removing the brain from the baſis of the ſcull, two ounces more of water were collected, that ſeemed to have been effuſed between the dura and pia mater. A ſmall quantity was alſo obtained from the theca vertebrarum, by holding the body with the head downwards.

Every part of the brain was in a healthy ſtate; as were alſo the thoracic and abdominal viſcera.

Of diſeaſes of this kind, ſubſequent to injuries of the head, there are, I believe, inſtances upon record; but as ſuch diſeaſes do not frequently occur, I flatter myſelf the caſe I have now communicated to you may not be deemed altogether uſeleſs.

The caſes which require the aſſiſtance of the trephine are, in general, clearly marked; but in the preſent inſtance the ſymptoms were not fuch as led me to confider them as arifing from blood or matter preffing on the encephalon.

*Golden Square,*

Nov. 26th, 1787.

